# A rare case of an appendiceal mass masquerading as a pelvic tumour and causing bilateral hydronephrosis

**DOI:** 10.2349/biij.8.2.e11

**Published:** 2012-04-01

**Authors:** SN Abdul Rashid, S Ab Hamid, S Mohamad Saini, R Muridan

**Affiliations:** 1 Department of Radiology, University of Malaya Medical Centre, Kuala Lumpur, Malaysia; 2 Department of Radiology, Faculty of Medicine and Health Sciences, Universiti Putra Malaysia, Selangor, Malaysia

**Keywords:** Appendiceal mass, bilateral hydronephrosis, pelvic teratoma, children

## Abstract

Diagnosing acute appendicitis in children can be difficult due to atypical presenting symptoms. While there are reported cases of acute appendicitis or appendiceal masses causing unilateral hydronephrosis, bilateral hydronephrosis as a complication of appendiceal mass is very rare. We report a case of a child who presented with cardinal symptomatology associated with the urogenital tract. Ultrasound (US) investigation showed a pelvic mass causing bilateral hydronephrosis. An initial diagnosis of a pelvic teratoma was made based on the US and computed tomography (CT) scan findings. The final diagnosis of an appendiceal mass causing bilateral hydronephrosis was established intraoperatively.

## INTRODUCTION

The appendix is a 2–20 cm part of the large bowel, arising from the caecum, 1.7–2.5 cm distal to the ileocaecal valve. The most common anatomical position of the appendix is retrocaecal (60–65%); followed by pelvic, where the tip lies in the lesser pelvic cavity (30%); subcaecal (0.5%); and ileocaecal (1.5%). In 5% of cases, the appendix is partially or totally located in the retroperitoneal space.

Appendicitis and appendicular abscesses are frequently found in paediatric populations. Urinary tract obstruction is an uncommon but well-recognized consequence of appendicitis. Obstruction usually occurs on the right side, and the occurrence of bilateral hydronephrosis secondary to acute appendicitis is very rare.

We report a case of a patient who presented with bilateral hydronephrosis, which was initially attributed to a pelvic teratoma. However, operative findings revealed an appendiceal mass.

## CASE REPORT

An eight-year-old Chinese girl presented to a private hospital with a nine-day history of suprapubic pain, frequency, dysuria and mild dehydration associated with low-grade fever. Her vital signs were stable and there was no palpable mass.

**Figure 1:**
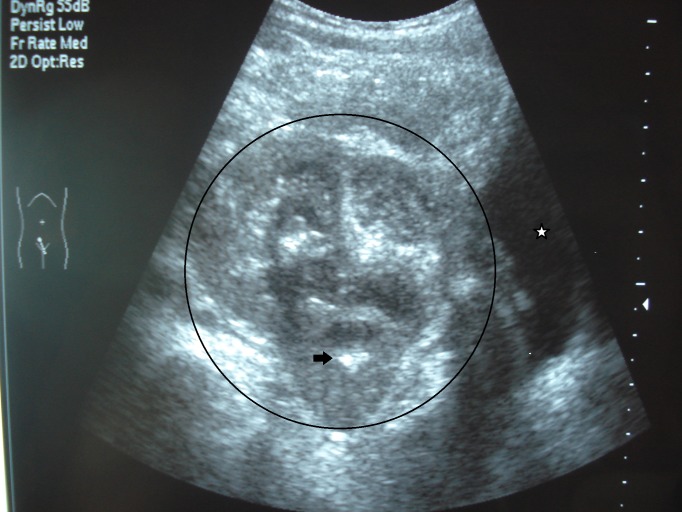
Ultrasound of the pelvic in axial section showing a heterogeneous mass (black circle) and calcification (black arrow) in close proximity to the urinary bladder (asterisk).

**Figure 2:**
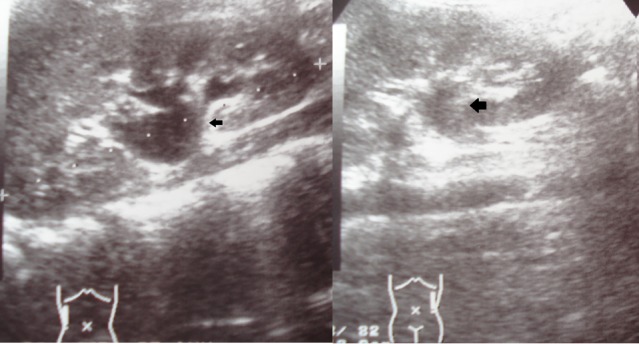
Ultrasound of the abdomen showing bilateral hydronephrosis (right more than left) as pointed by the black arrows.

Abdominal ultrasound showed a large, heterogenous mass in the pelvis measuring 6.2 cm × 5.0 cm × 6.1 cm, with cystic areas and calcification within it (Figure 1). Both kidneys were mildly hydronephrotic, predominantly on the right side (Figure 2). The urinary bladder was normal. There was no free fluid in the pelvis. The provisional diagnosis of a pelvic teratoma causing bilateral hydronephrosis was made. Blood investigation showed an elevated white cell count of 17.6 × 10^3^/L. Urinalysis showed the presence of ketones, trace amounts of protein and 1–2 (0–1/HPF) red blood cells. Renal profile was normal. She was given intravenous antibiotics and referred to our centre for a second opinion and further management.

In our centre, she was afebrile with gradually improving urinary symptoms. Physical examination revealed no mass per abdomen. The abdomen was non-tender. Human chorionic gonadotropin (hCG) and Alpha-fetoprotein (AFP) levels were normal.

In view of possible pelvic malignancy, a contrast-enhanced CT (SOMATOM SENSATION16, Siemens, Forchheim, Germany) of the thorax, abdomen and pelvis was done. The scan parameters were as follows: Helical, 80 KV, 200 mA, slice thickness of 5.0 mm, and 50 ml of contrast media was injected intravenously without oral or rectal contrast.

**Figure 3:**
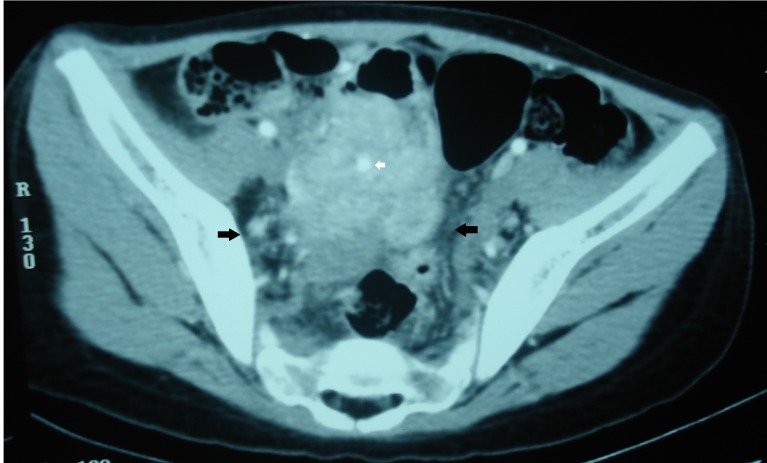
Contrast enhanced pelvic CT in axial section showing a heterogeneous mass in the pelvis with calcification (white arrow) and the surrounding bowels are poorly separated from this mass (black arrows).

A heterogeneous, enhancing, soft tissue mass was shown arising from the right side of the pelvis measuring 4.5 cm × 3.0 cm × 3.5 cm. Calcification was noted within it with no fat component and the surrounding bowel loops were poorly separated from the mass (Figure 3). The appendix was not visualised. There was minimal fluid in the Pouch of Douglas. The para-aortic and para-caval lymph nodes were not enlarged. The liver, pancreas and spleen were normal. There was very mild right-sided hydronephrosis, while the left kidney was normal. No abnormalities were noted within the thorax. The diagnosis of a possible intra-abdominal bowel-related mass instead of a pelvic malignancy was made based on the CT findings.

Laparotomy was performed, revealing a mass adherent to the bowel in the pelvis. Intra-operatively, an appendiceal mass due to a ruptured retroileal appendix was found. The appendix was situated at the retroileal region, inflamed, measuring 5.0 cm. The mass was removed and there were no immediate or late complications following surgery. Histology showed only inflammatory cells consistent with an appendiceal mass.

The patient was treated with intravenous metronidazole180 mg TDS, Gentamicin 180 mg OD and Cloxacillin 360 mg QID for four days. The patient was discharged well on oral antibiotics for one week.

Follow-up ultrasound examination showed a lesser degree of hydronephrosis on the right side and that the left kidney was normal. One year following surgery, both kidneys were normal.

## DISCUSSION

While acute appendicitis is a common condition affecting all age groups, it is more prevalent in childhood and early adolescence. It is the most common cause of emergency abdominal surgery in children and adolescents. The classical triad of abdominal pain, vomiting, and fever should always raise the question of appendicitis. Alvarado scoring is an established and proven method for the early diagnosis of acute appendicitis [1]. Based on her history, physical examination, and laboratory tests, the total score for this patient was six points, which was compatible with the diagnosis of acute appendicitis. However, the clinical presentation was very atypical.

Since the use of US by Puylaert in the diagnosis of acute appendicitis in 1986 [2], US has become the first line of diagnostic tools to detect or exclude acute appendicitis, especially in the paediatric patients. On US, the normal appendix has smooth and even hypoechoic bands, no demonstrable intraluminal exudates, an absence of periappendiceal fat infiltration, and an absence of blood flow in a thickened appendiceal wall [3]. Marked inflammatory changes of the surrounding mesentery or omentum and abnormal fluid collection or even an abscess formation in the right lower abdomen or pelvic cavity may be clues to the diagnosis of perforated appendicitis in children [3]. Wiersma *et al* reported that the sizes of the maximal outer diameter (MOD) and maximal mural thickness (MMT) in a normal appendix in children was 0.21–0.64 cm and 0.11–0.27 cm respectively [4]. Any measures exceeding these are considered abnormal.

It is recommended that when equivocal findings are noted on US, CT is the next modality of choice [5]. CT continues to be the most accurate imaging modality for diagnosing appendicitis [6]. Commonly practice utilises intravenous contrast with or without oral contrast or, less frequently, rectal contrast [7]. The most useful CT scan findings for the diagnosis of acute appendicitis are an enlarged appendix, appendiceal wall thickening, periappendiceal fat stranding and appendiceal wall enhancement [8]. Other findings include appendicolith, abcess formation, adenopathy, and the ‘arrowhead sign’ due to thickening of the caecal wall secondary to inflammatory changes from the appendicitis [9]. However, Rao *et al* noted that the same CT findings were present in both the acute and the recurrent or chronic appendicitis groups [9].

Overall accuracy for CT ranges from 88% to 98% [10] as compared to US, which has a lower sensitivity of 88% [11] and is highly operator-dependent. Although CT provides the highest accuracy for diagnosing appendicitis compared to US, radiation exposure for paediatric patients as well as exposure to contrast media needs to be considered seriously. Therefore, US remains the first-line imaging modality in the diagnosis of acute appendicitis. Furthermore, US is safe, easily accessible and, most importantly, does not use ionising radiation.

In this case, the decision for a contrast-enhanced CT of the thorax, abdomen, and pelvis was made to confirm the probable diagnosis of pelvic malignancy and other organ involvement. The typical findings of teratoma on CT are a complex mass containing fluid, fat, a fat-fluid level, and calcification [12]. However, other than the calcification, there were no other findings to suggest pelvic malignancy. The mass had a poor fat plane with the surrounding bowels. The appendix was not visualised both on US and CT. The calcification within the mass is most likely an appendicolith. The diagnosis of a possible intra-abdominal bowel related mass instead of pelvic malignancy was made based on the CT findings.

There are reported cases of pelvic malignancy masquerading as perforated appendicitis [13–14]. Conversely, there are also reported cases of inflammatory appendix masses masquerading as pelvic tumours in children [15]. This makes the diagnosis of appendiceal mass in children more challenging, not only for clinicians but also for radiologists. Consideration should be given to the possibility that an atypical pelvic mass with confusing clinical and radiological pictures in a child, particularly one containing calcification, may result from unrecognised chronic appendiceal inflammation [16]. In our case, the appendix was most likely not visualised both on US and CT because of its retroileal location, which is very rare.

Acute appendicitis remains a diagnostic challenge for the surgeon because the presentation is unclear in 20–35% of children, and increasing to 45% in adolescent females, as demonstrated in this case [17]. Since the urogenital tract is in close anatomical proximity to the appendix, the right distal ureter and the bladder are especially and frequently affected by the inflammatory reaction of acute appendicitis. This gives rise to various urogenital manifestations that can sometimes become the dominant symptomatology of appendicitis and its complications.

The atypical presentation of appendicitis as a disorder of the urinary tract, symptom of which include frequency, dysuria, anuria and urinary retention, is a relatively rare phenomenon. Urinary tract obstruction is an uncommon but well-recognised complication of appendicitis [18]. Ureteral obstruction typically occurs on the right side, with mild to moderate severity, due to localised periappendiceal inflammation [18]. The most likely explanation is the direct extension of the inflammatory process across the thin layer of the posterior parietal peritoneum, resulting in a segmental ureteral ileus similar to the bowel ileus seen with peritonitis. Bilateral ureteral obstruction is uncommon and usually due to mechanical obstruction by an appendiceal abscess [19]. There are reported cases of appendicitis presenting with urinary symptoms, including acute urinary retention [20–22].

The initial diagnosis of a pelvic malignancy instead of an appendicular mass was based on the atypical presentation, the radiological finding of a pelvic mass with calcification, and the findings of bilateral hydronephrosis, which are very atypical of acute appendicitis. This was initially thought to be a teratoma causing bilateral hydronephrosis. However, the patient’s hCG and AFP levels were normal. As the CT was performed three weeks after the initial presentation and the patient treated with antibiotics, the bilateral hydronephrosis had improved. Laporatomy was indicated in this case and confirmed the diagnosis of an appendiceal mass masquerading as a pelvic tumour and causing bilateral hydronephrosis.

In conclusion, the varying atypical anatomic deviations of the inflamed appendix can involve neighbouring structures, including the right ureter, the urinary bladder, and sometimes the left ureter, and thus give rise to acute symptoms that mimic urinary tract infection and/or obstruction [23]. Furthermore, acute or chronic appendicitis in children may also mimic pelvic malignancy clinically and radiologically. This should always be kept in mind by the radiologist and surgeon, in order to obtain the correct diagnosis and management.
